# Noncanonical cell death program independent of caspase activation cascade and necroptotic modules is elicited by loss of TGFβ-activated kinase 1

**DOI:** 10.1038/s41598-017-03112-1

**Published:** 2017-06-07

**Authors:** September R. Mihaly, Yosuke Sakamachi, Jun Ninomiya-Tsuji, Sho Morioka

**Affiliations:** 1 Department of Biological Sciences, North Carolina State University, Raleigh, NC 27695-7633 USA; 2 Present Address: Department of Microbiology, Immunology, and Cancer Biology, University of Virginia, Box 800734, Jordan Hall 7315, Charlottesville, VA 22908 USA

## Abstract

Programmed cell death (PCD) occurs in several forms including apoptosis and necroptosis. Apoptosis is executed by the activation of caspases, while necroptosis is dependent on the receptor interacting protein kinase 3 (RIPK3). Precise control of cell death is crucial for tissue homeostasis. Indeed, necroptosis is triggered by caspase inhibition to ensure cell death. Here we identified a previously uncharacterized cell death pathway regulated by TAK1, which is unexpectedly provoked by inhibition of caspase activity and necroptosis cascades. Ablation of TAK1 triggers spontaneous death in macrophages. Simultaneous inhibition of caspases and RIPK3 did not completely restore cell viability. Previous studies demonstrated that loss of TAK1 in fibroblasts causes TNF-induced apoptosis and that additional inhibition of caspase leads to necroptotic cell death. However, we surprisingly found that caspase and RIPK3 inhibitions do not completely suppress cell death in *Tak1*-deficient cells. Mechanistically, the execution of the third cell death pathway in *Tak1*-deficient macrophages and fibroblasts were mediated by RIPK1-dependent rapid accumulation of reactive oxygen species (ROS). Conversely, activation of RIPK1 was sufficient to induce cell death. Therefore, loss of TAK1 elicits noncanonical cell death which is mediated by RIPK1-induced oxidative stress upon caspase and necroptosis inhibition to further ensure induction of cell death.

## Introduction

Programmed cell death (PCD) is important for removal of damaged cells and is therefore indispensable for tissue homeostasis. Several forms of PCD exist including apoptosis, necroptosis, autophagy, pyroptosis and ferroptosis, which are triggered in different physiopathological contexts through distinct molecular machinery^1–3^. Apoptosis, one of the best characterized PCDs, executes cell death by activating intracellular cysteine protease caspases. During inflammation, apoptotic death is evoked by tumor necrosis factor receptor (TNFR) and Toll-like receptor (TLR) ligands, inducing an intracellular assembly of the death-inducing signaling complex (DISC) consisting of Fas-associated death domain (FADD), RIPK1, and an initiator caspase 8. Within the complex, caspase 8 is activated by self-cleavage to further activate downstream effector caspase 3 in order to execute apoptosis^1^. More recently, accumulating evidence suggests that necroptosis, at least in part, serves as a backup system to induce cell death when caspases are improperly inhibited or fail to be activated. For instance, when bacteria- and virus-derived molecules inhibit caspases, RIPK3 participates and plays a critical role in the DISC complex in which RIPK3 and RIPK1 phosphorylate and activate each other, resulting in the recruitment of another necroptotic mediator, mixed lineage kinase domain-like (MLKL)^4–6^. MLKL has been proposed to target several organelles such as mitochondria and the plasma membrane to govern cell death^7–11^. Therefore, RIPK3 activation is the key step in necroptosis and is recognized as a hallmark of necroptotic cell death^12, 13^. Moreover, in cancer cells, which often evade apoptotic death by overexpressing cell survival genes such as Bcl2 family proteins or cellular inhibitor of apoptosis (cIAP), RIPK3 is frequently mutated or down-regulated^14, 15^. However, under certain circumstances, both apoptotic and necroptotic pathways are concurrently inhibited by some virus-derived molecules^16–18^. Thus, it is highly possible that cells possess a further alternative pathway to ensure cell death under such conditions to maintain tissue homeostasis. Targeting such apoptotic- and necroptotic-independent cell death pathways can be powerful tools as therapeutic interventions.

TAK1 is a member of the mitogen-activated protein kinase kinase kinase (MAP3K) family and is a signaling intermediate of TNF and TLR signal transduction pathways leading to activation of transcription factor, NF-κB, as well as MAPKs^19, 20^. The activation of these downstream signaling factors produces cytokines while maintaining cell viability and lipid metabolism during inflammation^21, 22^. TAK1, similar to caspases and RIPK3, is known to be targeted and inhibited by pathogens^23^. Previous studies demonstrated that bone marrow-derived macrophages spontaneously undergo cell death upon TAK1 inhibition, but the precise mechanism of cell death has not been fully characterized^24–26^. In addition, recent studies have revealed that *Tak1* deficiency leads to TNF-dependent cell death in cultured fibroblasts and keratinocytes as well as *in vivo* epidermis and endothelium^27–29^. Mechanistically, *Tak1* deficiency induces profound activation of caspase 8 and 3, leading to apoptotic cell death. A pan caspase inhibitor ZVAD-FMK (zVAD) does not rescue the cell death in *Tak1*-deficient fibroblasts, suggesting that necroptotic cell death is alternatively activated. Consistently, further studies demonstrated that necrostatin-1 (Nec-1), one of the RIPK1 kinase inhibitors, blocks TNF- and zVAD-induced cell death in *Tak1*-deficient cells^25, 30^. However, recent studies reevaluated the role of RIPK1 in cell death induction, and it has been shown that RIPK1 kinase activity is not exclusively involved in necroptosis but is also involved in other types of cell death such as apoptosis and autophagic death^31–34^. Indeed, a previous study demonstrated that Nec-1 treatment suppresses caspase activation, suggesting activation of a RIPK1-caspase axis in *Tak1*-deficient cells^29^. Therefore, the contribution of RIPK1-RIPK3 necroptotic pathway in TNF- and zVAD-induced *Tak1*-deficient cell death is not fully characterized. In the current study, we unexpectedly found that *Tak1*-deficient macrophage death cannot be completely prevented by combined inhibition of caspase and RIPK3, suggesting that cells die though a previously uncharacterized mechanism. Furthermore, this noncanonical cell death pathway is triggered not only in macrophages but also in other cell types including fibroblasts. We further demonstrate that this pathway is still dependent on RIPK1, which triggers ROS accumulation to lead to oxidative stress-dependent cell death. Therefore, even when caspase and RIPK3 pathways are inhibited, RIPK1 ensures death-dependent elimination of cells by a noncanonical cell death program triggered by loss of TAK1.

## Results

### *Tak1* deletion induces macrophage necrosis

**Figure 1:**
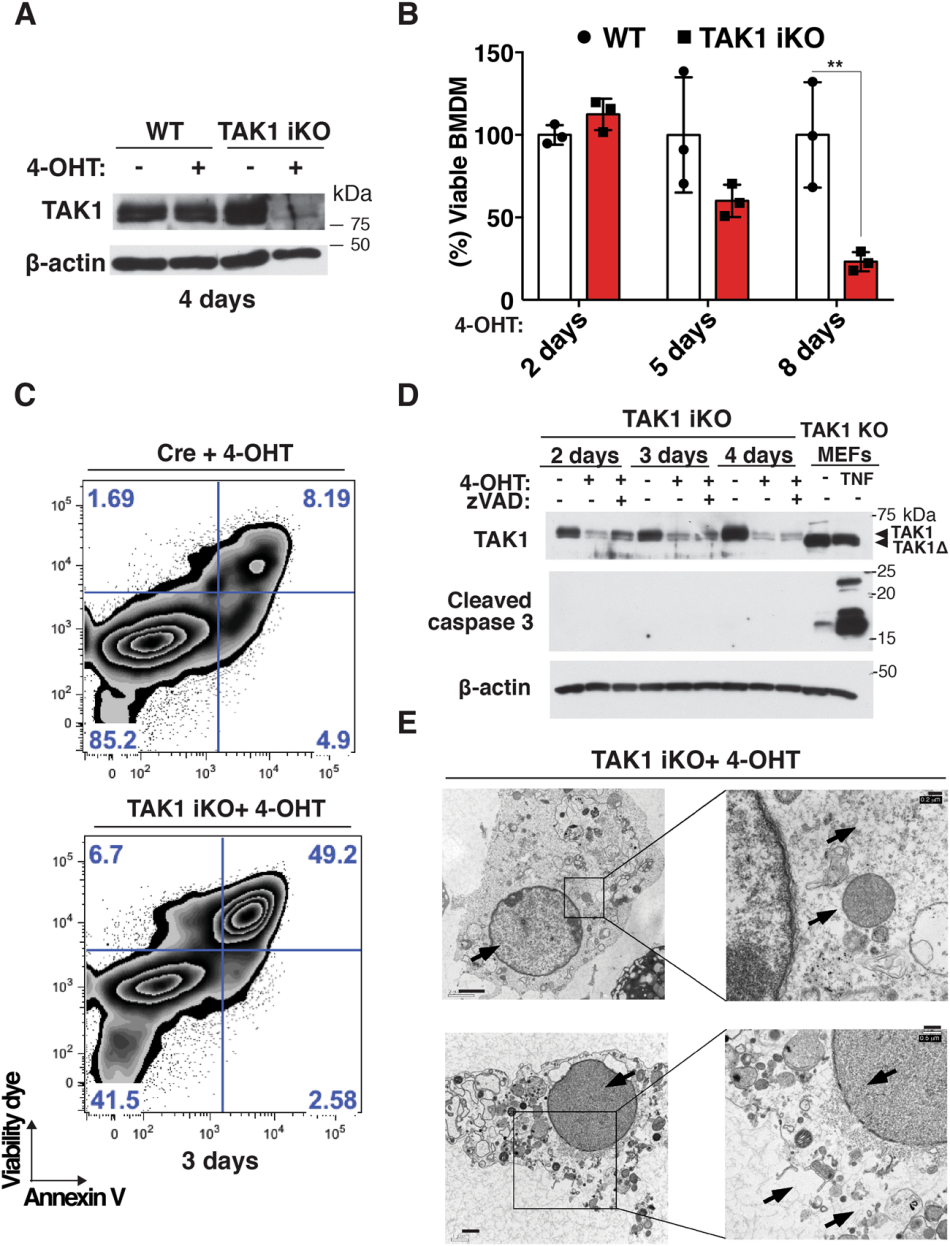
TAK1 deletion induces macrophage cell death with necrotic features (**A**) WT and TAK1^iKO^ BMDMs were treated with 0.3 µM 4-OHT or vehicle (ethanol) for 4 days. Expression of TAK1 was analyzed by immunoblotting. (**B**) WT and TAK1^iKO^ BMDMs were treated with 0.3 µM 4-OHT or vehicle for indicated days. Cell viability was measured by crystal violet assay. Data are the result of 3 independent experiments and show mean percentages of TAK1^iKO^ cell viability in each day +/− SD. **p-value < 0.01; two-way ANOVA (**C**) Cre-alone and TAK1^iKO^ BMDMs were treated with 0.3 μM 4-OHT for 3 days. Floating and attached cells were collected and stained with annexin V-Pacific Blue and Fixable viability dye eFlour 780, then analyzed on flow cytometer. The results shown are representative of 3 independent experiments. (**D**) WT and TAK1^iKO^ BMDMs were treated with 0.3 µM 4-OHT or vehicle (ethanol) together with or without 20 µM zVAD for indicated days. Caspase 3 activation was analyzed by anti-cleaved caspase 3 immunoblotting. Tak1-deficient fibroblasts (TAK1 KO) treated with 20 ng/ml TNF for 6 h was used as positive control for caspase 3 activation. (**E**) TAK1^iKO^ BMDMs at 5 days post 4-OHT treatment were analyzed using a transmission electron microscope. Scale bars, 2 μm (top left), 0.2 μm (top right), 1 μm (bottom left) or 0.5 μm (bottom right) as shown. Top images: Arrows indicate swollen mitochondria and enlarged nucleus and cytoplasm. Bottom images: Arrows show disrupted plasma membrane and perforated nuclear envelope and lack of nuclear condensation.

Bone marrow-derived macrophages (BMDMs) spontaneously die in culture when *Tak1* is deleted using a myeloid-specific gene deletion system driven by LysM-Cre^24–26^. In order to exclude the possibility that *Tak1-*deficient myeloid cells are being primed for cell death in the *in vivo* environment, and to further address the mechanism of the spontaneous cell death, we used an *in vitro* inducible *Tak1* knockout system (*Tak1* ^*flox/flox*^ *Rosa26.CreERT* referred to as TAK1^iKO^). We isolated bone marrow cells from *Tak1* ^*flox/flox*^ *Rosa26.CreERT* mouse and first differentiated them into mature BMDMs (Fig. S1A). BMDMs were then subsequently treated with 4-hydroxytamoxifen (4-OHT) to induce Cre-dependent gene deletion. The protein level of TAK1 was diminished within 2–4 days (Fig. 1A). Cell viability was concomitantly decreased with the reduction of TAK1, suggesting that loss of TAK1 *in vitro* is sufficient to trigger BMDM death (Fig. 1B). Acute expression of Cre is known to cause toxicity in bone marrow cells *in vivo* ^35^. In contrast, 4-OHT did not influence cell viability in *Tak1* ^*flox/+*^ *Rosa26.CreERT* BMDMs in culture (Fig. S1B), indicating that Cre expression or heterozygous deletion of *Tak1* does not affect macrophage mortality. Intriguingly, *Tak1*-deficient macrophages did not exhibit nuclear TUNEL staining, but instead, they displayed atypical cytoplasmic puncta staining (Fig. S2A). Macrophages are characterized by their high phagocytic activity^36^. Thus, this staining pattern might indicate that neighboring healthy macrophages cleared dying cells. In order to define the type of cell death observed in *Tak1*-deficient macrophages, we conducted Annexin V and cell permeability dye staining for the initial assessment of apoptosis and necrosis. Annexin V and cell permeability dye double-positive macrophages were increased by *Tak1* gene deletion at day 3, when cells start dying (Fig. 1C), suggesting that *Tak1* deletion causes necrosis or potentially apoptosis (secondary necrosis). To distinguish these, we monitored caspase 3 activation over 4 days by detecting cleaved caspase 3 (Figs 1D and S2B). Caspase 3 cleavage was not detected in *Tak1*-deficient macrophages, whereas TNF treatment induced profound caspase 3 activation in *Tak1*-deficient fibroblasts. Thus, *Tak1* deficiency is likely to induce a necrotic type of cell death in BMDMs. In line with this, transmission electron microscopy analysis revealed that *Tak1*-deficient macrophages exhibit morphological features of necrosis (e.g. cytoplasmic and organelle swelling, plasma membrane rupture), but not apoptotic features, such as nuclear condensation or apoptotic bodies (Fig. 1E). Collectively, *Tak1*-deficient macrophages die with more necrotic than apoptotic characteristics.

### RIPK1 induces *Tak1*-deficient macrophage death through a mechanism independent of caspase activity and RIPK3

**Figure 2:**
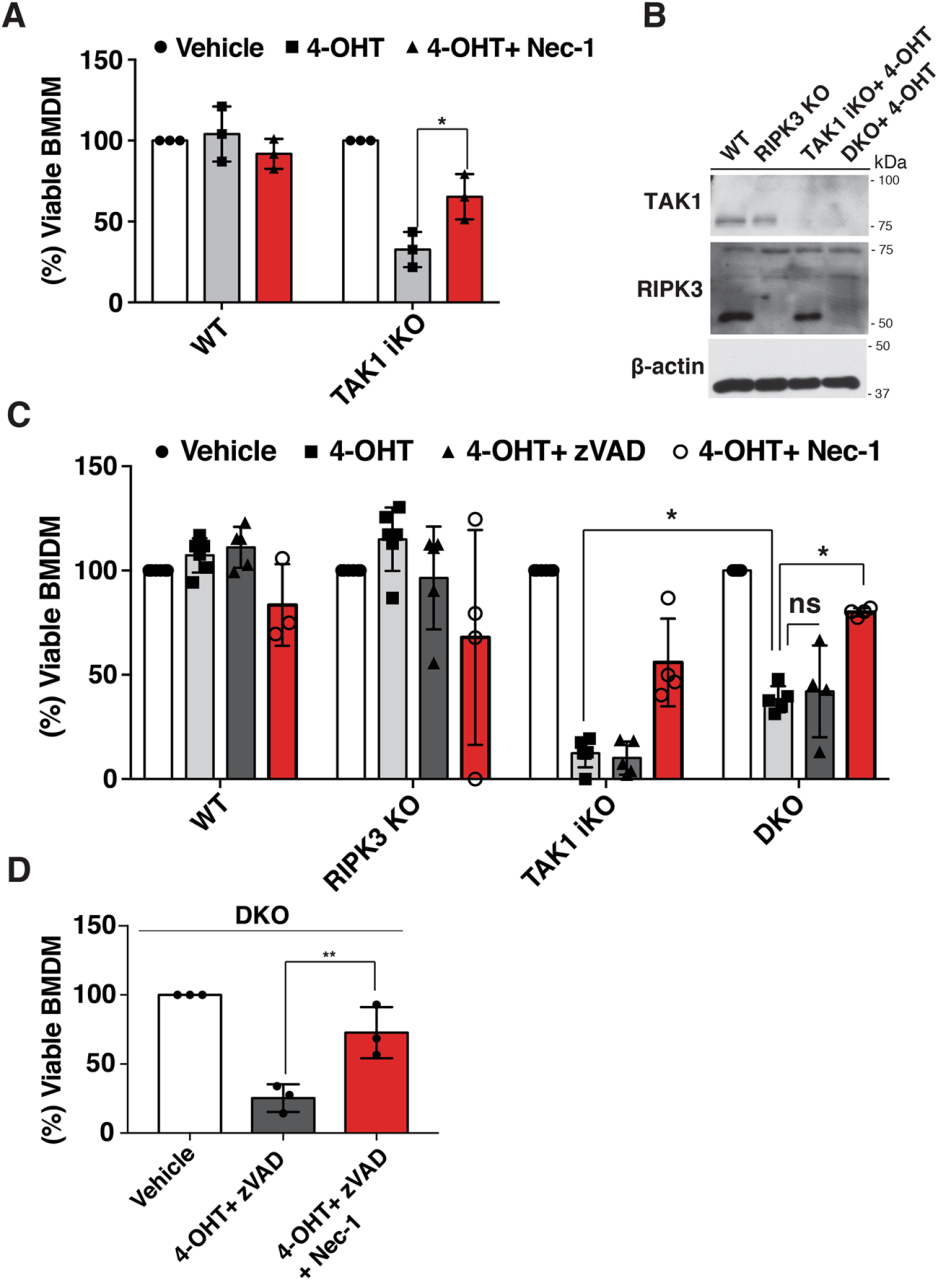
*Tak1*-deficient macrophage die through a caspase- and RIPK3- independent death pathway (**A**) WT and TAK1^iKO^ BMDMs cells were treated with 0.3 μM 4-OHT together with vehicle or Nec-1 for 7 days. Cell viability was determined by crystal violet assay. Data are the result of 3 independent experiments and show mean percentages +/− SD. *p-value < 0.05; two-way ANOVA. (**B**) Whole cell extracts were harvested from WT, TAK1^iKO^ and RIPK3 KO and DKO BMDMs treated with 0.3 µM 4-OHT for 4 days. (**C**) WT, *Ripk3* ^−/−^ (RIPK3 KO), TAK1^iKO^, and *Ripk3* ^−/−^ TAK1^iKO^ (DKO) BMDMs were treated with 0.3 μM 4-OHT or vehicle (ethanol) +/− 20 µM zVAD or 50 µM Nec-1. Data are the result of at least 3 independent experiments and show mean percentages +/− SD. ns, not significant; *p-value < 0.05; two-way ANOVA. (**D**) RIPK3 KO BMDMs were treated with 0.3 μM 4-OHT together with 20 µM zVAD and 50 µM Nec-1. Data are the result of 3 independent experiments and show mean percentages +/− SD. ns, not significant; **p-value < 0.01; one-way ANOVA.

Necrotic features observed in *Tak1*-deficient macrophages prompted us to address the possibility of necroptosis induction. We used necrostatin 1 (Nec-1), a well-established RIPK1 kinase inhibitor, and found that inhibition of RIPK1 effectively restored cell viability of *Tak1*-deficient macrophages (Fig. 2A). Notably, to our surprise, *Ripk3* deletion slightly restored the viability but did not completely rescue *Tak1*-deficient macrophage death. We still observed that over 60% of *Tak1-* and *Ripk3-*double-deficient macrophages underwent cell death (Fig. 2B and C). These suggest that although necroptosis has a minor role in inducing cell death, *Tak1*-deficient macrophages die predominantly through a necroptosis-independent mechanism. In addition, blocking both apoptosis and necroptosis by caspases inhibition and *Ripk3* deletion did not fully rescue cell death in *Tak1*-deficient macrophages, further suggesting the negligible involvement of caspase pathways (Fig. 2C). Surprisingly, inhibition of RIPK1 kinase activity by Nec-1 treatment significantly rescued the cell death in *Tak1-* and *Ripk3-*double-deficient BMDMs and *Tak1*/*Ripk3-* and caspase activity-deficient BMDMs (Fig. 2C and D). These results demonstrate that *Tak1*-deficient macrophages die by a previously uncharacterized mechanism, which is independent of caspase activity and RIPK3, and requires RIPK1 kinase activity.

In order to further confirm negligible involvement of necroptotic pathway and to explore the possible partner of RIPK1, we evaluated the involvement of MLKL. To this end, we used mouse-specific MLKL inhibitor, GW806742X. This compound, however, has been observed to potentially have mild RIPK1 inhibitory activity (unpublished observation)^37^. Therefore, we titrated the concentration of GW806742X. 50, 100 nM and 1 μM of GW806742X suppressed TNF-induced cell death in *Tab2*-deficient fibroblasts, which are known to undergo RIPK3-dependent necroptosis (Fig. S3A)^38^. In contrast, as discussed in the introduction, *Tak1*-deficient fibroblasts undergo RIPK1-dependent apoptosis. We treated cells with GW806742X and found that 1 μM of the compound significantly blocked TNF-induced cell death in *Tak1*-deficient fibroblasts, suggesting a potential effect on RIPK1 (Fig. S3A). Based on these results, we consider that 50–100 nM of GW806742X inhibits MLKL whereas 1 μM of the compound additionally inhibits RIPK1. We found that 50 and 100 nM of GW806742X did not rescue DKO BMDM cell death treated with 4-OHT + ZVAD, suggesting that RIPK1-dependent cell death does not require MLKL (Fig. S3A).

### RIPK1 induces Reactive Oxygen Species accumulation and death in *Tak1*-deficient macrophage

**Figure 3:**
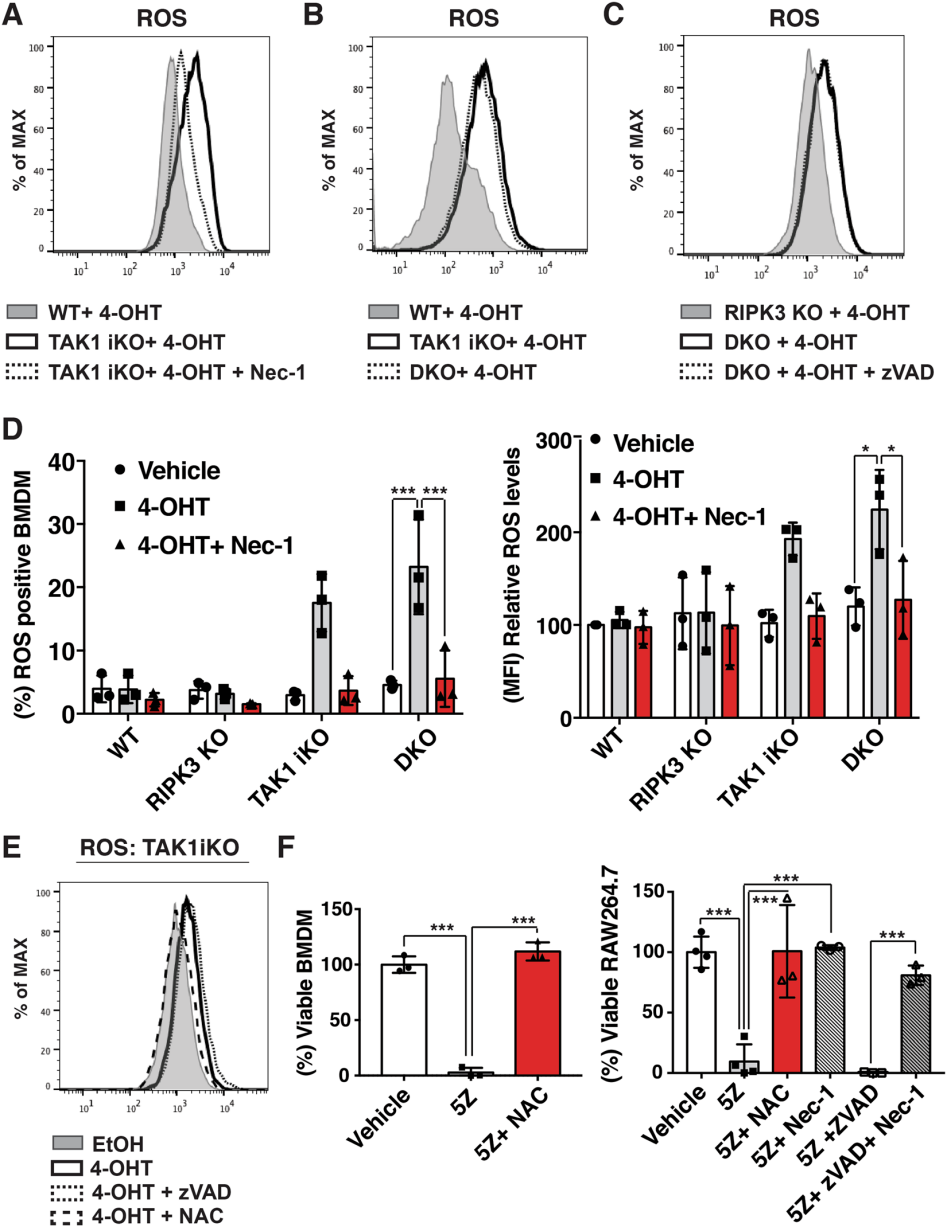
RIPK1 activity drives oxidative stress-induced cell death in *Tak1*-deficient macrophages through a caspase- and RIPK3- independent death pathway (**A**) WT and TAK1^iKO^ BMDMs were treated with 0.3 µM 4-OHT +/− 50 µM Nec-1 for 4 days, stained with CM-H_2_DCFDA and analyzed by flow cytometry. Cells were gated to exclude debris. The result shown is representative of three independent experiments (**B**) WT, TAK1^iKO^ and RIPK3 KO BMDMs were treated with 0.3 µM 4-OHT for 4 days. The result shown is representative of three independent experiments (**C**) RIPK3 KO and DKO were treated with 0.3 µM 4-OHT +/− 20 µM zVAD for 4 days. The result shown is representative of three independent experiments (**D**) WT, RIPK3 KO, TAK1^iKO^, and DKO BMDMs were treated with 0.3 μM 4-OHT or vehicle (ethanol) +/− 50 µM Nec-1. ROS positive BMDM and the relative ROS levels are shown. Data are the result of 3 independent experiments and show mean percentages +/− SD. *p-value < 0.05; ***p-value < 0.001; two-way ANOVA. (**E**) TAK1^iKO^ BMDMs were treated with 0.3 µM 4-OHT with or without 7.5 mM NAC or 20 µM zVAD for 4 days. Cells were gated to exclude debris. The result shown is representative of three independent experiments. (**F**) RAW264.7 and WT BMDMs were incubated with 200 nM 5Z-7-oxozeaenol (5Z) with or without 7.5 mM NAC, 20 µM zVAD and 50 µM Nec-1 for 24 h. Cell viability was measured by crystal violet assay. Data are the result of 3 independent experiments and show mean percentages +/− SD. ***p-value < 0.001; one-way ANOVA.

In an effort to determine how this pathway executes cell death, we found that loss of *Tak1* leads to accumulation of ROS in macrophages (Fig. 3A). In line with the results of Fig. 2, deletion of *Ripk3* and additional inhibition of caspase activity did not reduce the ROS accumulation (Fig. 3B and C). The percentage of ROS positive cells as well as the level of ROS accumulation were similarly increased in *Tak1-*deficient and *Tak1-* and *Ripk3-*double-deficient macrophages (Fig. 3D). Importantly, this ROS accumulation was almost completely blocked by Nec-1 treatment (Fig. 3A and D), suggesting that RIPK1 is the driver of oxidative stress. To determine if the ROS accumulation causes cell death in this context, we treated cells with a ROS scavenger N-acetyl cysteine (NAC). NAC treatment effectively reduced ROS (Fig. 3E), and partially restored the cell viability in *Tak1*-deficient macrophages although the effect was not striking in this setting (Fig. S3C). We hypothesized that long-term treatment with NAC would be toxic to macrophages over the time course. Therefore, we used a selective inhibitor of TAK1, 5Z-7-oxozeaenol, to acutely inhibit TAK1 kinase activity. 5Z-7-oxozeaenol effectively induced cell death in BMDMs within 24 h as well as in the macrophage cell line, Raw264.7. Cell death was completely rescued by NAC or Nec-1 treatment (Fig. 3F). These results demonstrate that ablation of TAK1 induces macrophage death through RIPK1-dependent ROS accumulation. To further elucidate the mechanism of RIPK1-dependent oxidative stress-induced cell death, we looked at the expression level of antioxidant genes in the absence or presence of Nec-1. We found that *Tak1* deletion alone reduced the expression of superoxide dismutase 2 (SOD2) and glutathione S-transferase A2 (GSTA2), and that these expression levels were not rescued by Nec-1 (Fig. S4A). We anticipate that RIPK1 actively produces ROS, through the mechanism remains elusive, and that loss of TAK1 reduces the ability to scavenge for generated ROS. Although TAK1-dependent antioxidant gene regulation has been previously reported^39^, the mechanism of how RIPK1-derived ROS execute cell death is elusive. It was previously reported that ROS-dependent JNK activation induces death^40^. Thus, we have looked at the activity of JNK in DKO BMDM in the absence or presence of Nec-1 and found that TAK1 deletion rather slightly reduced phosphorylation of JNK and that RIPK1 inhibition did not affect the status of JNK activity (Fig. S4B). Furthermore, inhibition of JNK did not change the accumulation of ROS to the same extent as cell death (Fig. S4C,D). Collectively, unlike published observation where ROS accumulation leads to JNK-dependent cell death in some circumstance, this data implies that the JNK pathway is not likely to be involved in the induction of cell death in TAK1, RIPK3, caspase inhibited BMDMs.

Finally, we further investigated the trigger of RIPK1-dependent cell death pathway. As TNF has been previously demonstrated to induce cell death in *Tak1*-single-deficient BMDMs, we used anti-TNFα antibody. TNF neutralizing antibody successfully rescued cell death in zVAD-treated TAK1/RIPK3 DKO BMDMs and zVAD-treated RAW264.7 (Fig. S4E). Therefore, RIPK1-dependent cell death is triggered by the TNF signaling pathway.

### TNF induces a noncanonical cell death pathway independent of caspase activity and RIPK3 in *Tak1*-deficient fibroblasts

**Figure 4:**
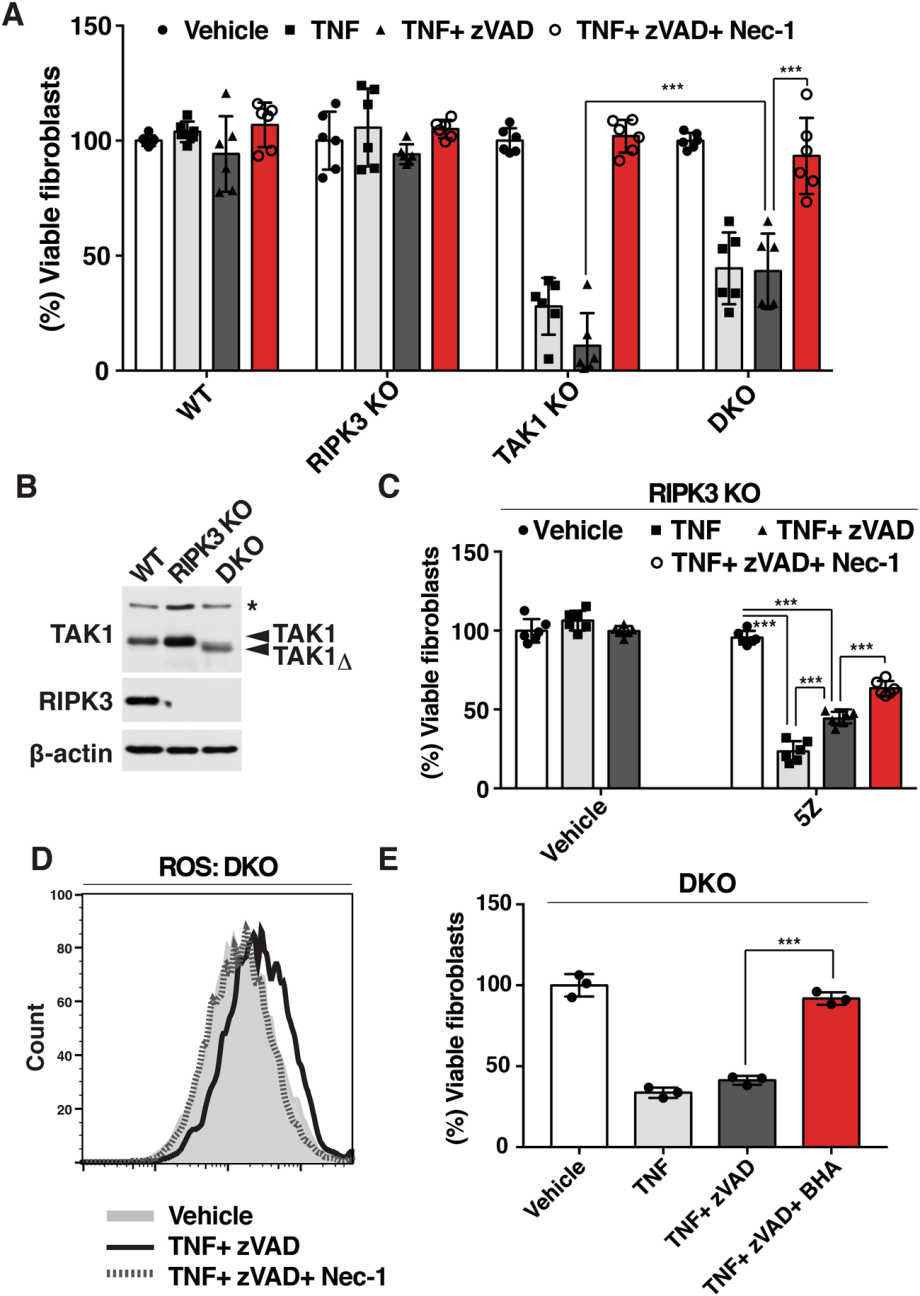
TNF induces RIPK1-dependent noncanonical cell death independent of caspase activity and necroptosis in the absence of TAK1 (**A**) WT, *Tak1*-deficient (TAK1 KO), *Ripk3* ^−/−^ (RIPK3 KO) and *Tak1*-deficient Ripk3−/− (DKO) were pre-treated with vehicle (DMSO) or 20 μM zVAD +/− 50 µM Nec-1 for 1 h and stimulated with 20 ng/ml TNF for 24 h. Cell viability was determined by crystal violet assay. Data are the result of 3 independent experiments and show mean percentages +/− SD. NS, not significant; ***p-value < 0.001; two-way ANOVA. (**B**) WT, RIPK3 KO and DKO fibroblasts were analyzed by immunoblotting. *Tak1*-deficient fibroblasts expressed a truncated nonfunctional form of TAK1 (TAK1Δ). β-actin is shown as a loading control. (**C**) RIPK3 KO fibroblasts were pretreated with 20 μM zVAD, 50 µM Nec-1 and/or 5Z-7-oxozeaenol for 1 h and stimulated with 20 ng/ml TNF for 24 h. Data are the result of at least 3 independent experiments and show mean percentages +/− SD. NS, not significant; ***p-value < 0.001; one-way ANOVA. (**D**) DKO fibroblast were pretreated with 20 µM zVAD and/or 50 µM Nec-1 for 1 h and stimulated with 20 ng/ml TNF. ROS were analyzed by CM-H_2_DCFDA at 6 h post TNF stimulation. The result shown is representative result of 3 independent experiments. (**E**) DKO fibroblasts were pre treated with 100 µM BHA and/or 20 µM zVAD for 1 h and stimulated with 20 ng/ml TNF for 24 h. Cell viability was determined by crystal violet assay. Data are the result of 3 independent experiments and show mean percentages +/− SD. ***p-value < 0.001; one-way ANOVA.

Unlike macrophages, *Tak1*-deficient fibroblasts are viable but sensitive to TNF-induced apoptosis^29^. In order to investigate whether caspase- and RIPK3-independent cell death is conserved in fibroblasts, we analyzed a *Tak1-deficient* fibroblast model. One could anticipate that when apoptosis is inhibited in *Tak1*-deficient fibroblasts, TNF-induced apoptosis may shift to RIPK3-dependent necroptosis. Therefore, we asked whether TNF and zVAD induced cell death can be rescued by *Ripk3* deletion (Fig. 4A and B). TNF successfully induced cell death in *Tak1*-deficient fibroblasts, which was not rescued by zVAD. To our surprise, and consistent with the results of *Tak1-*deficient macrophages, *Ripk3* deletion did not completely rescue cell death induced by TNF and zVAD in *Tak1*-deficient fibroblasts (Fig. 4A). We confirmed the effect of zVAD on caspase activities (Fig. S5). These suggest either that TNF and zVAD induce a non-necroptotic (RIPK3-independent) cell death in *Tak1*-deficient fibroblasts or that combined inhibition of caspase and RIPK3 further shift the cell death pathway to another death cascade in the absence of TAK1. Additional inhibition of RIPK1 greatly rescued the cell death suggesting that this previously uncharacterized cell death pathway is also RIPK1-dependent and conserved in fibroblasts (Fig. 4A). We further tested the effect of TAK1 inhibition by treating fibroblasts with 5Z-7-oxozeaenol. Combined inhibition of TAK1 and caspases induced TNF-mediated fibroblast death, which was rescued by Nec-1 (Fig. 4C). Consistent with the observation in macrophages, ROS were increased in *Tak1*-deficient fibroblasts following TNF stimulation in the presence of zVAD and *Ripk3* deletion, and restored by Nec-1 treatment (Fig. 4D). Another ROS scavenger, butylated hydroxyanisole (BHA), effectively rescued TNF- and zVAD-induced cell death in *Tak1-* and *Ripk3*-double-deficient fibroblasts (Fig. 4E). These results further demonstrate that *Tak1* deletion and caspase inhibition generally induces a previously uncharacterized type of cell death that is RIPK3-independent and RIPK1-ROS axis-dependent.

### RIPK1 induces ROS accumulation independent of caspase and RIPK3

**Figure 5:**
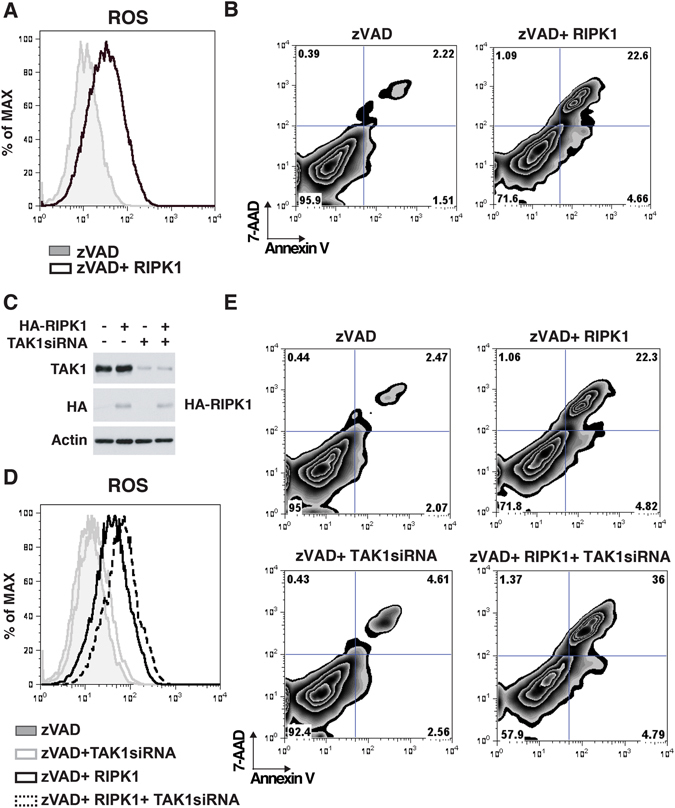
RIPK1 induces ROS accumulation and cell death in the absence of RIPK3 and caspase activities. (**A** and **B**) HeLa cells were transfected with an expression vector for GFP-tagged RIPK1 or GFP together with 20 µM zVAD, incubated for 24 h, and stained with CellROX dye or Annexin V-APC and 7-AAD, then analyzed on a flow cytometer. (**C**) Proteins expression of TAK1 and RIPK1 were analyzed by immunoblotting. β-actin is shown as a loading control. (**D** and **E**) HeLa cells were transfected with siRNA targeted against TAK1 gene, incubated for 24 h and then transfected with an expression vector for GFP-tagged RIPK1 or GFP together with 20 µM zVAD, incubated for another 24 h, and stained with CellROX dye or Annexin V-APC and 7-AAD, then analyzed on a flow cytometer.

Finally, as RIPK1 was found to be crucial for induction of the cell death, we hypothesized that RIPK1 alone may be sufficient to induce ROS accumulation. To test this, we overexpressed RIPK1 in HeLa cells, which do not express RIPK3^14^. Overexpression of RIPK1 increased ROS accumulation as well as Annexin V- and 7-Aminoactinomycin D (7-AAD)-positive necrotic cells in the presence of Z-VAD (Fig. 5A and B). In order to investigate the involvement of TAK1 in this pathway, we knocked down TAK1 expression by siRNA (Fig. 5C). In RIPK1 overexpressing cells, knockdown of TAK1 further facilitated RIPK1-dependent ROS accumulation, which led to greater induction of necrosis (Figs 5D,E and S6C). These results were recapitulated by using another siRNA against TAK1 (Fig. S6). Collectively, these results demonstrate that RIPK1 is sufficient to induce ROS accumulation and cell death even in the absence of RIPK3 and caspase activity and that TAK1 prevents RIPK1-dependent ROS accumulation and cell death.

## Discussion

*Tak1* deletion primarily induces apoptosis upon TNF treatment in several cell types^29, 41^. However, even if apoptosis is blocked by inhibition of caspases, *Tak1* deficiency is known to still result in cell death^29^. Because this cell death was blocked by ablation of RIPK1, it was previously recognized as necroptosis^25, 30^. However, RIPK1 is not exclusively important for necroptosis and is also involved in other types of cell death including apoptosis^31–33^. In contrast to RIPK1, RIPK3 has thus far been recognized as a critical mediator of necroptosis^4, 12^. We demonstrate here that even when both caspases and RIPK3 are inactive, *Tak1* deficiency still induces cell death in fibroblasts and macrophages. Thus, this cell death is not likely to be the previously defined necroptosis. We note here that Lamothe *et al*. reported that knockdown of *Ripk3* completely blocked cell death in *Tak1*-deficient fibroblast with caspase inhibition, which is somewhat inconsistent with our results and may due to the TNF-treatment time span^25^. Although the cause of this inconsistency has yet to be solved, our results clearly demonstrate that complete abrogation of RIPK3 using a genetic deletion system does not completely rescue cell death in *Tak1*-deficient fibroblasts and macrophages. We conclude that *Tak1* deficiency induces a noncanonical cell death which is dependent on RIPK1 and subsequent ROS accumulation when caspases and RIPK3 are inhibited. As necroptotic pathway serves as back-up pathway when apoptosis fails to be activated, TAK1-dependent cell death pathway can be further back-up system when both apoptosis and necroptosis are inactivated. Therefore, this pathway may serve as a crucial host defense system against some viruses, which can target both apoptotic and necroptotic machinery. In addition, TAK1 inhibition can be a powerful tool to treat cancer cells that have developed resistance to apoptotic and necroptotic insult. Indeed, several studies have suggested that TAK1 inhibition successfully kills some types of cancer cells^39, 42, 43^.

The current study suggests that noncanonical RIPK1-dependent cell death does not require MLKL, since an MLKL inhibitor did not block cell death (Fig. S3A). However, in order to eliminate potential off-target effects, investigating noncanonical RIPK1-depending cell death using an *Mlkl* gene deletion model will further support this conclusion. In addition, although the cell death research field has taken advantage of well-established inhibitors for cell death molecules, the involvement of any given protein and its activity have to be carefully interpreted. For instance, *Ripk1* gene deletion sensitizes cells to TNF-induced apoptosis, whereas RIPK1 kinase inhibition does not induce apoptosis but rather inhibits apoptosis or necroptosis^44^. As another example, RIPK3 kinase dead knock-in mouse induces apoptosis, whereas *Ripk3* deletion does not trigger cell death, but rather inhibits necroptotic cell death^45^. In our current study, we used zVAD to inhibit caspase activity, however, the involvement of caspase in the noncanonical cell death pathway would be further dissected using both zVAD and caspase deletion models, which warrant further studies.

We show that ROS are causally associated with this cell death. We have previously demonstrated that *Tak1* single deletion induces RIPK1-dependent ROS accumulation, leading to caspase activation and apoptotic cell death in response to TNF^38, 41, 43^. The modes and mechanisms of RIPK1-dependent ROS accumulation in *Tak1-deficient* cell and in TAK1, caspases and RIPK3 inhibited cell might differ as the latter pathway lacks caspase activation and caspase activation has been shown to induce ROS^46^. Nevertheless, how does RIPK1 upregulate ROS? Several mechanisms have been implicated in ROS accumulation leading to cell death. In the context of necroptosis, modulation of mitochondrial function, in that a protein complex RIPK1-RIPK3ß impairs mitochondrial fusion/fission, could cause ROS accumulation^5, 7, 47^. Other potential mechanisms include alterations of energy metabolism^48^. In the current study, ROS are increased even in the absence of *Ripk3* in *Tak1*-deficient cells. RIPK1 and RIPK3 have structural similarities in that they have signature amino acid motifs, named RIP homotypic interaction motifs (RHIM), which form amyloid fibrils to execute cell death^49^. RIPK1 expression alone, although to a slightly lesser extent than RIPK1-RIPK3 co-expression, is able to make this amyloid fibril^49^. Therefore, it is possible that RIPK1 utilizes similar machinery to induce ROS accumulation in TAK1, caspase and RIPK3 inhibited cells. It has long been known that overexpression of RIPK1 induces cell death^50, 51^, but these results were not further mechanistically investigated. Some of these early experiments used RIPK3-deficient cells such as HeLa cells to test the effect of overexpression of RIPK1. We now demonstrate that RIPK1 overexpression is indeed capable of inducing ROS accumulation and cell death even in the absence of caspase activation and RIPK3. Therefore, future studies investigating the mechanism of RIPK1-induced cell death are of considerable interest and could provide an important clue for elucidating a new type of cell death program. Another important question is how TAK1 modulates RIPK1 activity in the context of cell death induction. We recently showed that TAK1 actively mediates necroptosis while TAK1 is a major inhibitor of apoptosis^29^. TAK1 and RIPK1 physically interact both directly and through polyubiquitin chains^52, 53^. Thus, trans-modifications and the mode of binding between TAK1 and RIPK1 may be critically involved in the regulation of cell death. Our current results warrant further studies delineating functional interactions between RIPK1 and TAK1 in cell death signaling pathways.

## Materials and Methods

### Bone Marrow Derived Macrophages and Fibroblasts

C57BL/6 mice with *Tak1* ^*flox/flox*^ were described previously^54^. *Rosa26.CreERT* (Jax mice, B6;129-Gt(ROSA)26Sortm1(cre/ERT)Nat/J) and *Ripk3* ^−/−^^55^ mice were bred in our facility to produce the genotypes used for later experiments. Bone marrow cells from mice having *Tak1*, *Rosa26.CreERT*, and *RIP3* ^*−/−*^ mutations, alone or in combination, were isolated and cultured in 70% cell media (DMEM + 10% BGS + 1% Pen/Strep + 1% Amphotericin B) + 30% L929-conditioned media for 3 days with wash and new media on day 2. Cells having *Rosa26.CreERT* were treated with 0.3 µM 4-hydroxytamoxifen (4-OHT) for 2–4 days to achieve gene deletion. All animal research experiments were conducted with the approval of the North Carolina State University Institutional Animal Care and Use Committee (IACUC). *Tak1*-deficient and *Tak1-* and *Ripk3*-double-deficient fibroblasts were described previously^29^.

### Reagents, Antibodies, Plasmids and siRNA

Specific monoclonal and polyclonal antibodies against the following antigens were used: β-actin (Sigma), caspase 3 (Cell Signaling), cleaved caspase 3 (Cell Signaling), RIPK3 (Sigma-Aldrich), JNK (Santa Cruz), P-JNK (Cell Signaling), mouse TNFα neutralizing D2H4 (Cell Signaling) and TAK1 (described previously) [56]. N-acetyl-L-cysteine (NAC) and butylated hydroxyanisole (BHA) (Sigma-Aldrich) were used to inhibit ROS generation. The RIPK1 inhibitor, Nec-1 (Nec-1) and pan-caspase inhibitor, zVAD-FMK (zVAD) were purchased from Enzo Life Sciences. The JNK inhibitor, SP600125, was purchased from SIGMA. The MLKL inhibitor, GW806742X, was purchased from SYNkinase. The TAK1 kinase inhibitor, 5Z-7-oxozeaenol (5Z) was described previously^56^. GFP- and HA-tagged RIPK1 (pEGFP-N1-GFP-RIP1) were a gift from Dr. Chan (University of Massachusetts Medical School) (Addgene plasmids)^57^. Two different TAK1 gene and non-targeting control siRNAs were generated (Tak1 siRNA, 5′-UCUCCUUGUAGUCGAUCUC; Tak1 siRNA #2, 5′-UCCAGAUUCACUCUGUUGCUUUGCC; and Non-targeting siRNA, 5′-UUCUCCGAACGUGUCACGU-3′). Fibroblasts were transfected with the siRNAs using TrasIT-X2 (Mirus).

### Quantitative RT–PCR

Total RNA was extracted from cells using RNeasy Mini Kit (Qiagen) and cDNA was synthesized using QuantiTect Reverse Transcription Kit (Qiagen) according to manufacturers’ instructions. Quantitative gene expression for mouse *Gsta2*, *Sod2* or housekeeping mouse *Gapdh* was performed using Taqman probes (Applied Biosystems) using StepOnePlus Real Time PCR System (ABI).

### Crystal Violet Assay

BMDMs were plated onto 12-well plates at a concentration of 2 × 10^5^ cells per well and treated with 0.3 µM 4-OHT for 2 days and N-acetyl cysteine, zVAD, and/or Nec-1 for 1–2 additional days. In experiments involving RAW cells, cells were pre-treated with inhibitors and subsequently treated with 5Z-7-oxozeaenol. Cells were fixed using 10% formalin, and stained with 0.1% crystal violet solution. The dye was eluted and analyzed at 595 nm.

### Flow Cytometry

BMDMs were detached from culture dishes and incubated with annexin V-Pacific Blue (BioLegend) or annexin V-APC (BD biosciences) and Fixable Viability Dye eFluor 780 (eBiosicence) or 7-AAD (Invitrogen) for cell death analysis, with CM-H_2_DCFDA (Invitrogen) or CellROX deep red staining kit (Thermo Fisher Scientific) for analysis of reactive oxygen species, or with antibodies (CD11b [M1/70], and; F4/80 [BioLegend]) for differentiation analysis. Fluorescence was detected by flow cytometry (BD Biosciences LSR II), and data were analyzed using FlowJo software (Tree Star). Events were gated to exclude dead cells and debris, then gated on FITC wave length (CM-H_2_ DCFDA), APC (CellROX deep red), PE-Cy5 (F4/80), Pacific Blue (CDllb or annexin V), or APC-Cy7 (fixable viability dye) when compared to unstained control.

### Western Blotting

BMDMs were lysed in extraction buffer (20 mM HEPES [pH 7.4], 150 mM NaCl, 12.5 mM β-glycerophosphate, 1.5 mM MgCl_2_, 2 mM EGTA, 10 mM NaF, 2 mM DTT, 1 mM Na_3_VO_4_, 1 mM PMSF, 20 µM aprotinin, 0.5% Triton X-100) and incubated on ice for 15 minutes. Cells and debris were then pelleted by centrifugation at 20,000 G for 10 min at 4**°**C. Cell extracts were resolved using SDS-PAGE and transferred to Hybond-P membranes (GE Healthcare). The membranes were immunoblotted with the indicated antibodies, and the bound antibodies were visualized with horseradish peroxidase-conjugated antibodies against rabbit or mouse IgG using the ECL Western blotting system (GE Healthcare).

### TUNEL Assay

Terminal deoxynucleotidyl transferase dUTP nick end labeling (TUNEL) assay was used to detect DNA fragmentation associated with apoptosis (Promega). BMDMs were grown on slides and treated with 0.3 µM 4-OHT for 4 days to achieve gene deletion. Slides were fixed in 4% formaldehyde in PBS and permeabilized in 0.2% Triton X-100 in PBS. DNA terminal nucleic acids were labeled with rTDT enzyme plus nucleotide mix and counterstained with DAPI. Cells were analyzed by fluorescence microscopy.

### Electron Microscopy

BMDMs were detached from culture dishes, washed once in PBS, fixed with McDowell Trump’s fixative, and embedded in 3% water agar. Ultrathin sections were captured with Philips Transmission Electron Microscope 208S using a Gatan Erlengshen ES1000W digital camera, and analyzed for signs of apoptosis or necrosis.

### Caspase Assay

Cell extracts were mixed with Caspase-Glo substrate mix (Promega) for the measurement of caspase-8 or caspase-3 activities according to the manufacturer’s instructions.

### Statistical Analysis

The means of two groups were compared using two-tailed unpaired Student’s t test. When three groups were compared, we used a one-way ANOVA test. When there were two variables, we used a two-way ANOVA test, followed by Tukey post hoc test to compare pairs of means.

All experiments described above were performed in accordance with relevant and approved guidelines and regulations.

## Electronic supplementary material

Supplemental Figures
