# Erratum: Noncanonical cell death program independent of caspase activation cascade and necroptotic modules is elicited by loss of TGFβ-activated kinase 1

**DOI:** 10.1038/s41598-017-09609-z

**Published:** 2017-09-06

**Authors:** September R. Mihaly, Yosuke Sakamachi, Jun Ninomiya-Tsuji, Sho Morioka

**Affiliations:** 1 Department of Biological Sciences, North Carolina State University, Raleigh, NC 27695-7633 USA; 2 Present Address: Department of Microbiology, Immunology, and Cancer Biology, University of Virginia, Box 800734, Jordan Hall 7315, Charlottesville, VA 22908 USA

*Scientific Reports* **7**:2918; doi:10.1038/s41598-017-03112-1; Article published online 07 June 2017

The original version of this Article contained an error in the title of the paper, where the word “Noncanonical” was incorrectly given as “Noncanocial”. This error has now been corrected in the HTML and PDF versions of this Article, and the Supplementary Information file that accompanies the Article.

