# Supplementary material for: Noncanonical cell death program independent of caspase activation cascade and necroptotic modules is elicited by loss of TGFβ-activated kinase 1
Source: Sci Rep. 2017 Jun 7;7:2918. doi: 10.1038/s41598-017-03112-1 (PMC5462742; doi:10.1038/s41598-017-03112-1)
Supplement: Supplementary file 1 — Supplemental Figures [file 41598_2017_3112_MOESM1_ESM.pdf]

Noncanonical cell death program independent of caspase activation cascade and necroptotic modules is elicited by loss of TGFβ-activated kinase 1

September R. Mihaly[1,3], Yosuke Sakamachi[1], Jun Ninomiya-Tsuji[1], Sho Morioka[1,2,3,4]

Affiliations:

[1]Department of Biological Sciences, North Carolina State University, Raleigh, NC 27695-7633, USA; Tel: 01-919-513-1586; Fax: 01-515-7169.

[2]Current address: Department of Microbiology, Immunology, and Cancer Biology, University of Virginia, Box 800734, Jordan Hall 7315, Charlottesville, VA 22908, USA

[3]These authors contributed equally to this work

[4]To whom correspondence should be addressed: Sho Morioka

Email address: sm9ss@virginia.edu

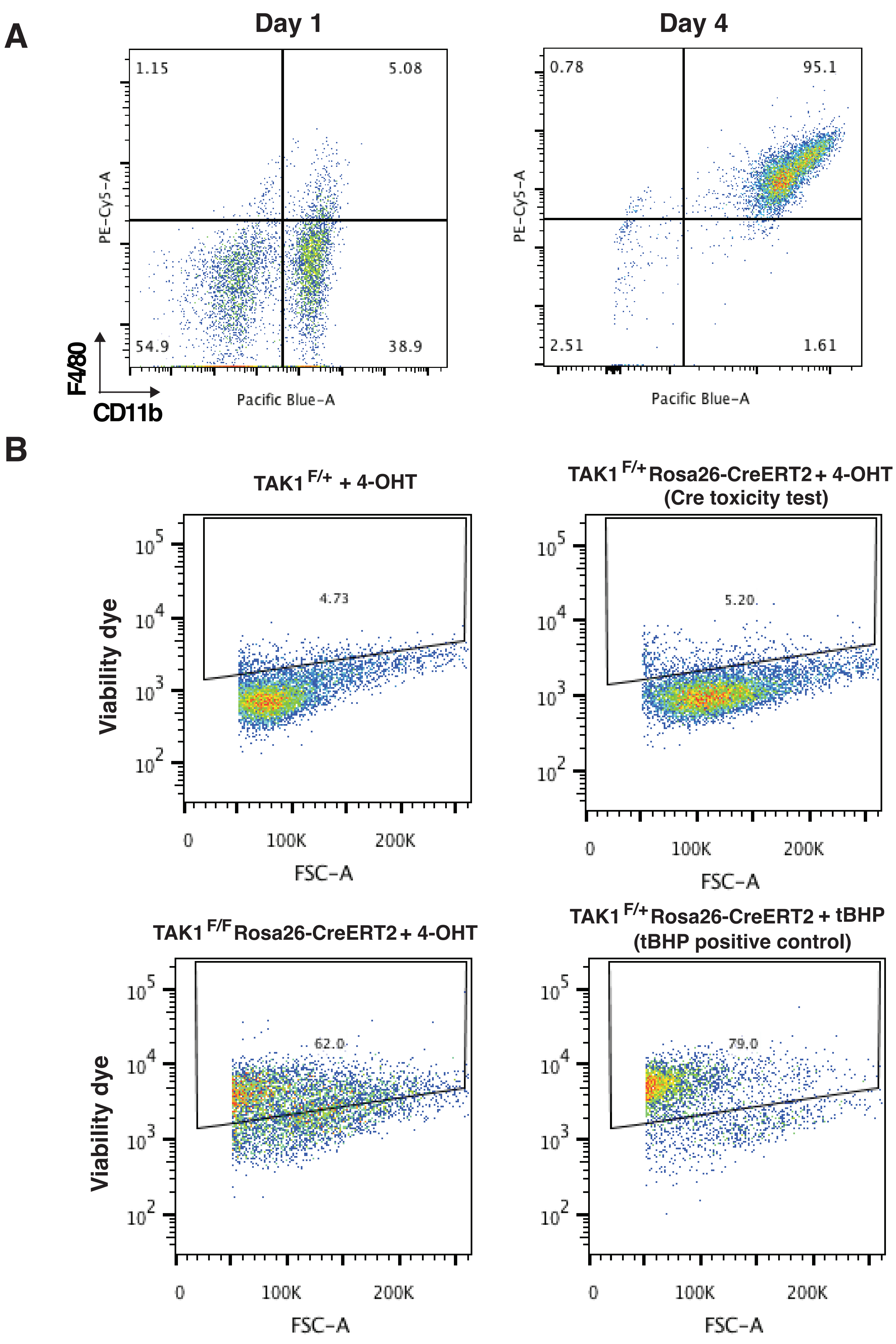
A
Day 1
Day 4
1.15
5.08
54.9
38.9
0.78
95.1
2.51
1.61
PE-Cy5-A
Pacific Blue-A
F4/80
CD11b
B
TAK1 F/+ + 4-OHT
TAK1 F/+ Rosa26-CreERT2 + 4-OHT
(Cre toxicity test)
TAK1 F/F Rosa26-CreERT2 + 4-OHT
TAK1 F/+ Rosa26-CreERT2 + tBHP
(tBHP positive control)
4.73
5.20
62.0
79.0
Viability dye
FSC-A
0
100K
200K

A

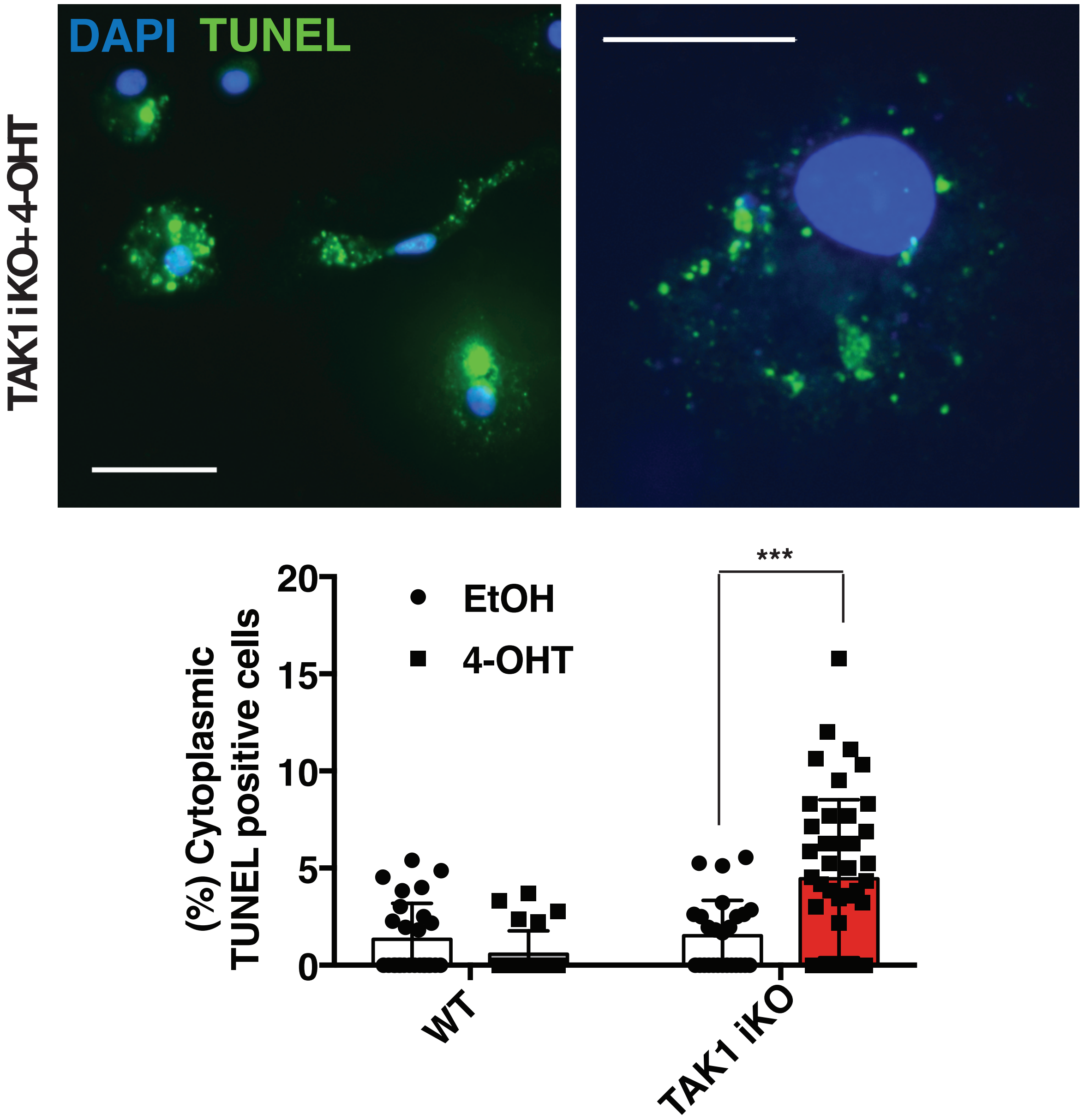

DAPI TUNEL
TAK1 iKO+4-OHT
***
EtOH
4-OHT
(%) Cytoplasmic TUNEL positive cells
20
15
10
5
0
WT
TAK1 iKO

B

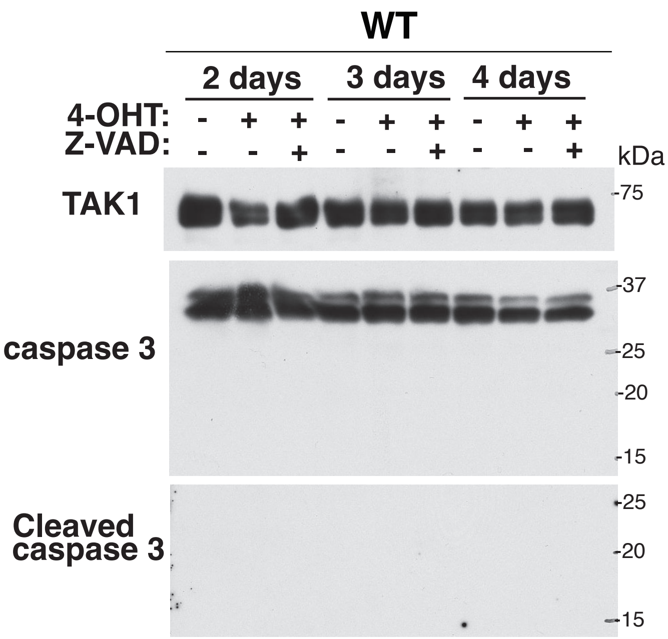

WT
2 days
3 days
4 days
4-OHT: - + + - + + - + +
Z-VAD: - - + - - + - - +
kDa
TAK1
caspase 3
Cleaved caspase 3
75
37
25
20
15
25
20
15

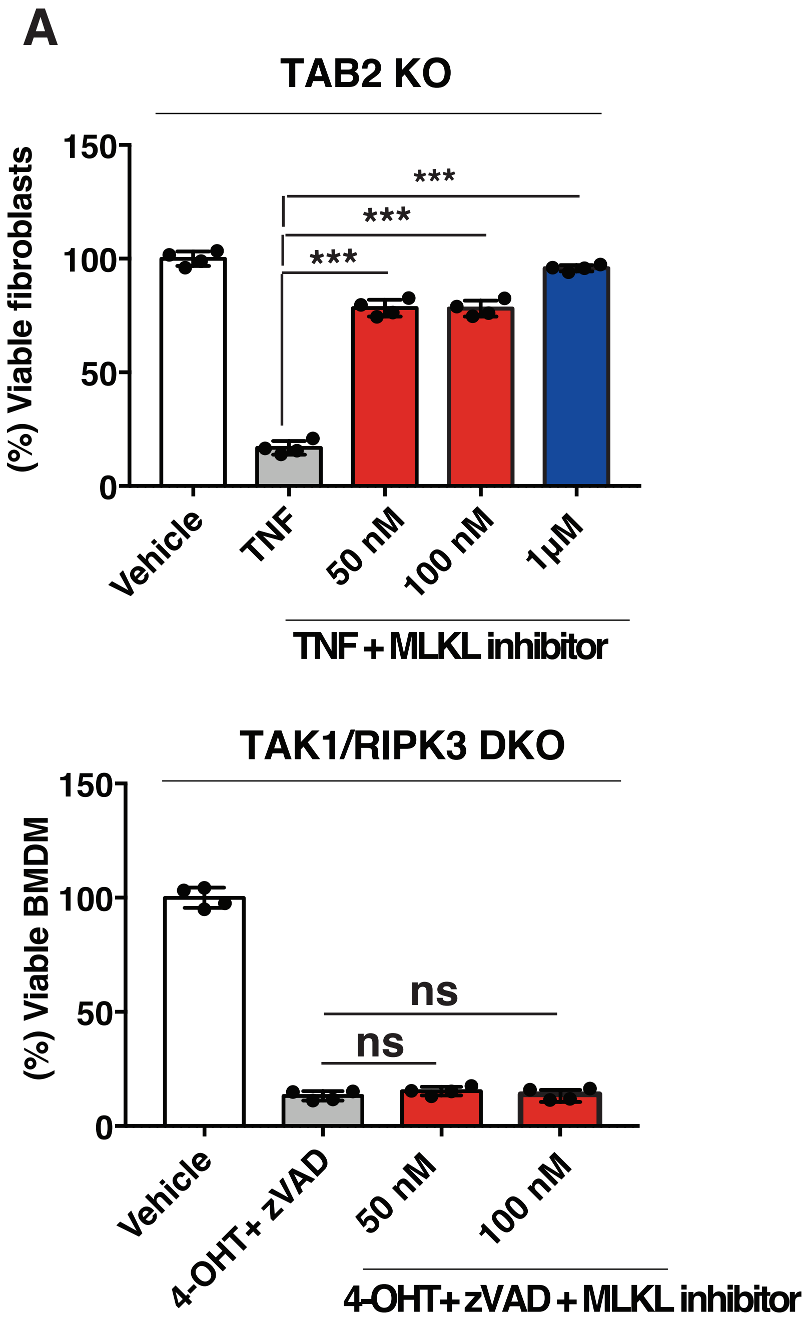
A
TAB2 KO
(%) Viable fibroblasts
150
100
50
0
***
***
***
Vehicle
TNF
50 nM
100 nM
1μM
TNF + MLKL inhibitor
TAK1 KO
(%) Viable fibroblasts
150
100
50
0
***
ns
ns
Vehicle
TNF
50 nM
100 nM
1μM
TNF + MLKL inhibitor
TAK1/RIPK3 DKO
(%) Viable BMDM
150
100
50
0
ns
ns
Vehicle
4-OHT+ zVAD
50 nM
100 nM
4-OHT+ zVAD + MLKL inhibitor
B
TAK1 iKO
(%) Viable BMDM
150
100
50
0
*
Vehicle
4-OHT
4-OHT+ NAC

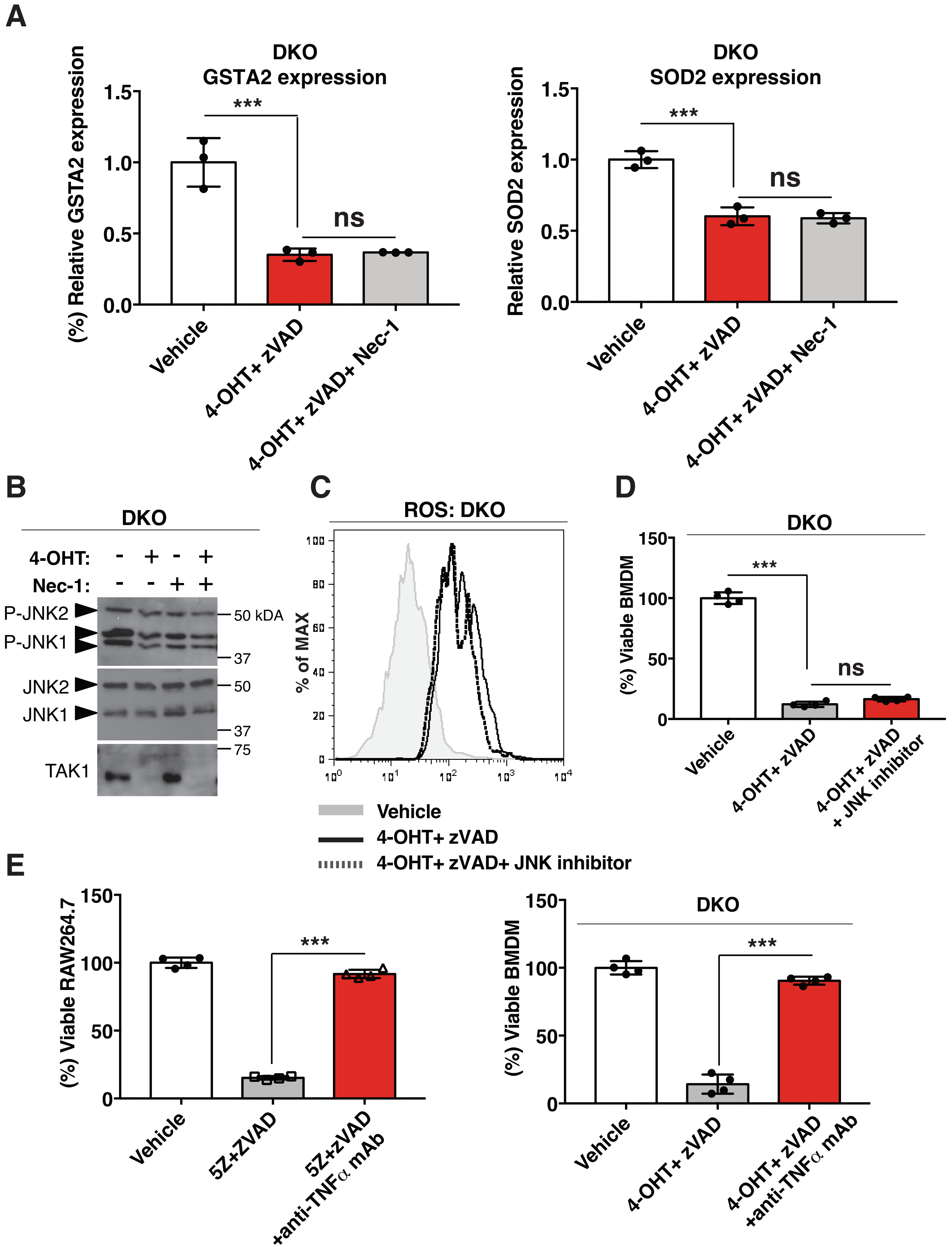
A
DKO
GSTA2 expression
(%) Relative GSTA2 expression
1.5
1.0
0.5
0.0
***
ns
Vehicle
4-OHT+ zVAD
4-OHT+ zVAD+ Nec-1
DKO
SOD2 expression
Relative SOD2 expression
1.5
1.0
0.5
0.0
***
ns
Vehicle
4-OHT+ zVAD
4-OHT+ zVAD+ Nec-1
B
DKO
4-OHT: - + - +
Nec-1: - - + +
P-JNK2
P-JNK1
JNK2
JNK1
TAK1
50 kDA
37
50
37
75
C
ROS: DKO
% of MAX
Vehicle
4-OHT+ zVAD
4-OHT+ zVAD+ JNK inhibitor
D
DKO
(%) Viable BMDM
150
100
50
0
***
ns
Vehicle
4-OHT+ zVAD
4-OHT+ zVAD + JNK inhibitor
E
(%) Viable RAW264.7
150
100
50
0
***
Vehicle
5Z+ZVAD
5Z+ZVAD +anti-TNFα mAb
DKO
(%) Viable BMDM
150
100
50
0
***
Vehicle
4-OHT+ zVAD
4-OHT+ zVAD +anti-TNFα mAb

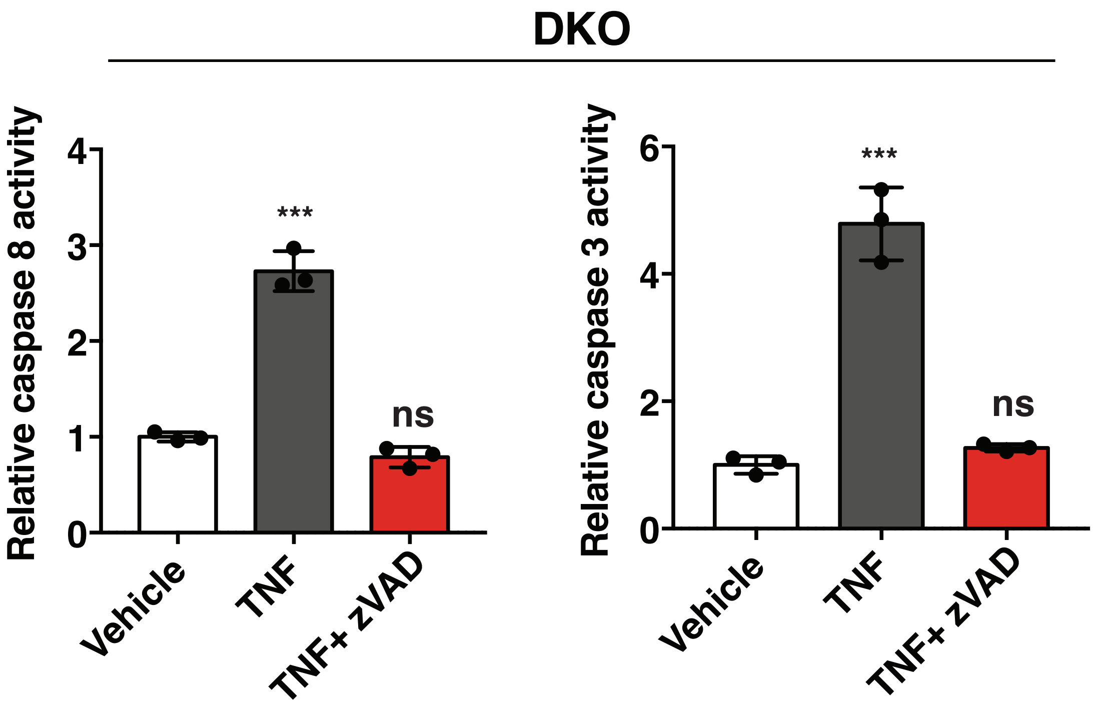
DKO
Relative caspase 8 activity
0
1
2
3
4
***
ns
Vehicle
TNF
TNF+ zVAD
Relative caspase 3 activity
0
2
4
6
***
ns
Vehicle
TNF
TNF+ zVAD

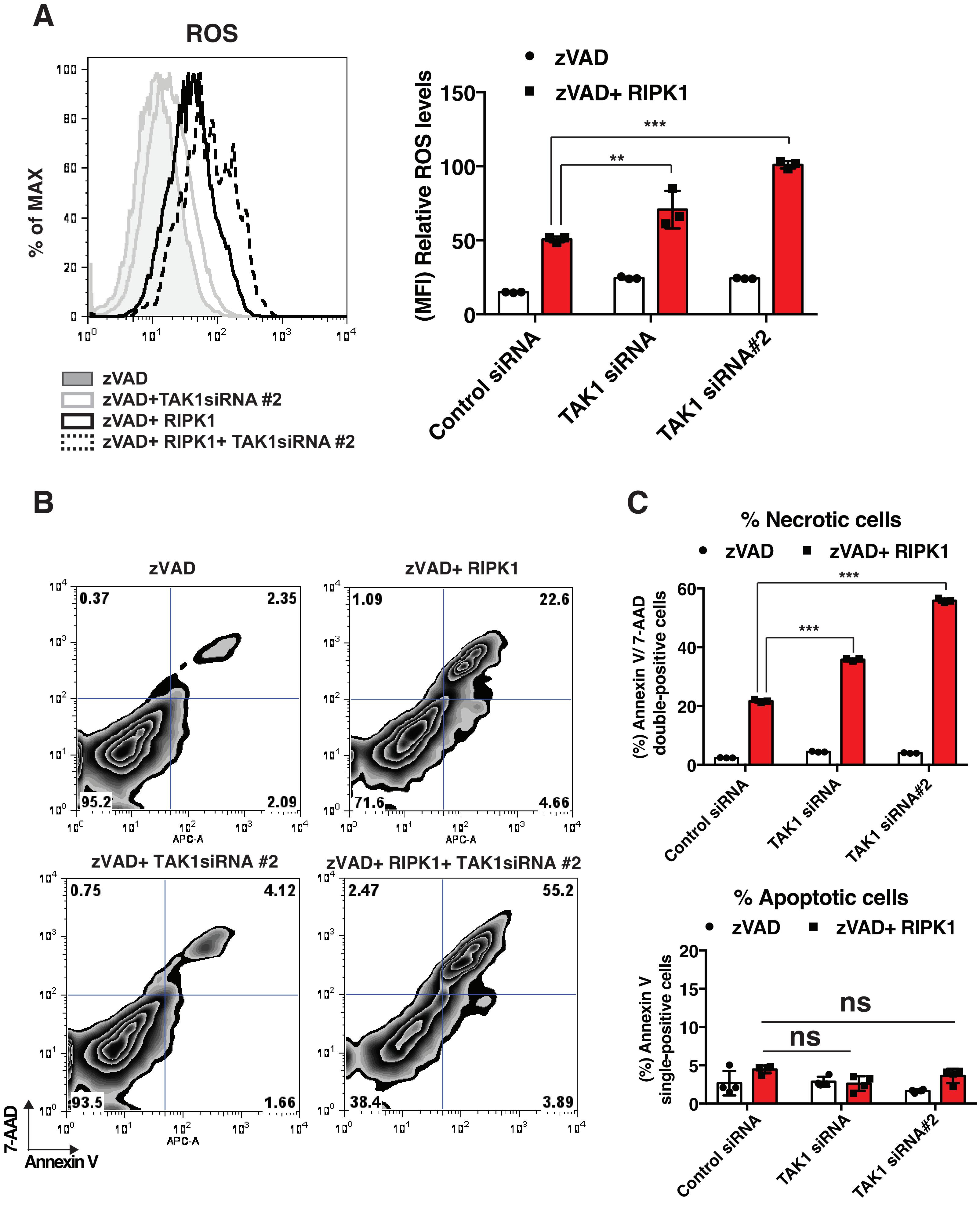
A
ROS
% of MAX
zVAD
zVAD+TAK1siRNA #2
zVAD+ RIPK1
zVAD+ RIPK1+ TAK1siRNA #2
zVAD
zVAD+ RIPK1
(MFI) Relative ROS levels
***
**
Control siRNA
TAK1 siRNA
TAK1 siRNA#2
B
zVAD
zVAD+ RIPK1
0.37
2.35
95.2
2.09
1.09
22.6
71.6
4.66
APC-A
zVAD+ TAK1siRNA #2
zVAD+ RIPK1+ TAK1siRNA #2
0.75
4.12
93.5
1.66
2.47
55.2
38.4
3.89
7-AAD
Annexin V
C
% Necrotic cells
zVAD
zVAD+ RIPK1
(%) Annexin V/ 7-AAD double-positive cells
***
***
Control siRNA
TAK1 siRNA
TAK1 siRNA#2
% Apoptotic cells
zVAD
zVAD+ RIPK1
(%) Annexin V single-positive cells
ns
ns
Control siRNA
TAK1 siRNA
TAK1 siRNA#2

Supplemental Figure Legends

Figure S1. *In vitro* characterization of BMDMs

(A) Freshly isolated bone marrow cells and BMDMs (L929-conditioned culture for 4 days) were incubated with F4/80 and CD11b antibodies and analyzed by flow cytometry. Figure shown is representative of three different experiments.

(B) Cells from mice with the indicated genotypes were incubated with fixable viability dye (Live/Dead) and quantified on a flow cytometer. Percent positive for Live/Dead is shown. Cells were treated with 1 mM tBHP for 1 h as a positive control (bottom right).

Figure S2. Characterization of *Tak1*-deficient BMDM death

(A) TUNEL shows unusual pattern of cytoplasmic puncta staining. TAK1^{iKO} and control (WT) BMDMs were cultured 4 day with 0.3 μM 4-OHT. Cells were fixed, permeabilized, and labeled with fluorescein-12-dUTP and counterstained with DAPI. Scale bars, 20 μm (left panel) and 10 μM (right panel). Representative images of TUNEL staining are shown. Graph shows the percent of TUNEL positive cells per 1000 cells counted. Each dot shows a percentage of TUNEL positive cells per area (approximately 40 cells per area).

(B) Western blotting for TAK1, caspase 3 and cleaved caspase 3 in WT BMDMs treated with 0.3 μM 4-OHT and 20 μM zVAD for 2, 3 or 4 days.

Figure S3. Oxidative stress but not RIPK3 or MLKL is responsible for *Tak1*-deficient BMDM death

(A) *Tab2*-deficient (TAB2 KO) and *Tak1*-deficient (TAK1 KO) fibroblasts were pre-treated with vehicle (DMSO) or indicated concentration of GW806742X for 1 h and stimulated with 20 ng/ml TNF for 24 h. DKO BMDMs were treated with 0.3 µM 4-OHT for 2 days, then subsequently treated with 20 μM zVAD and GW806742X for additional 2 days. Cell viability was determined by crystal violet assay. Data are the result of 3 independent experiments and show mean percentages +/- SD. ns, not significant; ***p-value < 0.001; one-way ANOVA.

(B) TAK1^{iKO} BMDMs were treated 0.3 μM 4-OHT for 2 days and subsequently treated with 500 μM NAC for additional 2 days. Viability was assessed by crystal violet assay. Figure shown are mean percent viable +/- SD. * p-value < 0.05; one-way ANOVA.

Figure S4. Investigation of the mechanism of *Tak1*-deficient BMDM death

(A) DKO BMDMs were treated with 0.3 μM 4-OHT +/- 50 μM Nec-1 for 4 days and RNA was extracted. Relative expressions of GSTA2 and SOD2 are shown. Figure shown are mean +/- SD. ns, not significant; *** p-value < 0.001; one-way ANOVA.

(B) Western blotting for TAK1, JNK and P-JNK in DKO BMDMs treated with 0.3 μM 4-OHT and 20 μM zVAD for 4 days.

(C and D) DKO BMDMs were treated with 0.3 μM 4-OHT for 2 days, then subsequently treated with 20 μM zVAD and 20 μM SP600125 for additional 2 days. ROS was detected by CellROX dye and cell viability was determined by crystal violet assay. Figure shown are mean percentages +/- SD. ns, not significant; ***p-value < 0.001; one-way ANOVA.

(E) RAW264.7 cells were incubated with 200 nM 5Z-7-oxozeaenol (5Z) with or without 10 μg/ml of anti-TNFα neutralizing antibody and 20 μM zVAD for 24h. DKO BMDMs were treated with 0.3 μM 4-OHT for 2 days, then subsequently treated with 20 μM zVAD and 10 μg/ml of anti-TNFα neutralizing antibody for additional 2 days. Cell viability was measured by crystal violet assay. Data are the result of 3 independent experiments and show mean percentages +/- SD. *** p-value < 0.001; one-way ANOVA.

Figure S5. The effect of zVAD on caspase 3 and 8 activities in DKO fibroblasts treated with TNF

DKO fibroblasts were pre-treated with (DMSO) or 20 μM zVAD for 1 h and stimulated with 20 ng/ml TNF for 6 h. Caspase activities relative to vehicle samples in cellular extracts are shown. Data are the result of 3 independent

experiments and show mean percentages +/- SD. ns, not significant; *** p-value < 0.001; one-way ANOVA.

Figure S6. The effect of TAK1 inhibition on RIPK1-induced ROS accumulation and necrosis induction

(A and B) HeLa cells were transfected with another siRNA targeted against TAK1 gene, incubated for 24 h and then transfected with an expression vector for GFP-tagged RIPK1 or GFP together with 20 μM zVAD, incubated for another 24 h, and stained with CellROX dye or Annexin V-APC and 7-AAD, then analyzed on a flow cytometer.

(C) Percentage of necrotic cells (Annexin V/ 7-AAD double-positive cells and percentage of apoptotic cells (Annexin V single-positive cells are shown. The results shown are representative of three different experiments and show mean percentages +/- SD. ns, not significant; *** p-value < 0.001; two-way ANOVA.
